# Insulin Autoimmune Syndrome in a 25-Year-Old, Previously Healthy Kuwaiti Man

**DOI:** 10.1155/2019/8919457

**Published:** 2019-12-12

**Authors:** Einas Alrashidi, Thamer Alessa

**Affiliations:** ^1^Department of Internal Medicine, Al-Farwaniya Hospital, Kuwait; ^2^Division of Endocrinology, Diabetes and Metabolism, Jaber Al-Ahmad Hospital, Kuwait

## Abstract

Insulin autoimmune syndrome (IAS) is a disease characterized by hyperinsulinaemic hypoglycaemia associated with autoantibodies against endogenous insulin. We have described a case of a 25-year-old, previously healthy Kuwaiti man who was admitted to the Mubarak Al-Kabeer hospital with a history of recurrent hypoglycaemia. The patient revealed that he had taken several different injectable anabolic steroids and growth hormone with oral amino acids and other tablets (fat burners) for bodybuilding in the last two months. He denied knowingly using insulin or insulin analogues. The patient had elevated fasting insulin level (>301 uIU/mL) and elevated insulin autoantibodies (>100.0 IU/mL). After appropriate work-up, he was diagnosed with IAS. After treatment with prednisolone (1 mg/kg/day), the patient had complete recovery. In patients with repeated hypoglycaemia, IAS should be considered in the differential diagnosis. Glucocorticoid therapy can be effective for the treatment of hypoglycaemia in patients with IAS.

## 1. Case Report

A 25-year-old, previously healthy Kuwaiti man was brought by ambulance to the Mubarak Al-Kabeer Hospital in May 2014 after he became unconscious. A capillary glucose value of <2 mmol/L was measured by the emergency medical service. He was managed in the emergency department with intravenous 5% dextrose infusion and recovered with no sequelae, after which he was admitted to the medical ward for further investigation.

The patient mentioned a 2-months history of recurrent episodes of palpitation, dizziness, light-headedness, and sweating and had recurrent visits to the emergency room in April 2014 with documented values of hypoglycaemia (capillary glucose value reached as low as 3.5 mmol/L) which improved after receiving intravenous dextrose infusion. Those symptoms developed usually late after meals and after prolonged hours with no carbohydrate intake and improved after eating. The episodes never happened immediately postprandial. There were no signs of seizures. The symptoms also improved after transient use of oral dexamethasone in high doses daily for several days in mid-April 2014 which were prescribed by an endocrinologist empirically to treat possible adrenal insufficiency. After stopping the dexamethasone, the hypoglycaemic episodes recurred.

The patient was previously healthy but did report an uncertain amount of weight gain and also a history of erectile dysfunction for 1 month that was not investigated and had not been treated. He had no previous surgeries. There was no family history of insulinoma or any other significant disease. The patient revealed that he took several different injectable anabolic steroids and growth hormone together with oral amino acids, and other tablets (fat burners) for bodybuilding in the previous two months. He denied knowingly using insulin or insulin analogues. The patient is single with 4 pack-year smoking history and works in a private company.

## 2. Investigations

In the medical ward, he still required 5% dextrose infusion and then 10% to maintain normoglycaemia; however, several additional but less frequent hypoglycaemic episodes occurred. On admission, his weight was 66 kg, height was 170 cm, body mass index was as 22.83 kg/m^2^, blood pressure was 125/75 mm Hg, and pulse rate was 65 beats per minute. The physical examination showed a nonpalpable thyroid and no features of hyperpigmentation. Testicular exam revealed normal size testes and phallus with normal hair distribution.

Urine sulfonylurea screen was not available at our hospital. When intravenous dextrose infusion was held, the patient was kept fasting and under close observation in the ward with plasma glucose measured repeatedly using glucose meter (Abbott Diabetes care Ltd, range road, Witney, Oxon, UK), and after 24 hours of fasting in a planned 72 hours fasting test to assess the hypoglycaemic event, the patient had a hypoglycaemic attack with symptoms of dizziness, palpitation, and sweating. The capillary glucose value was 2.5 mmol/L. and several laboratory blood tests to assess the hypoglycaemic event was performed (Table 1).

During the hypoglycaemia, serum insulin was elevated (>301 uIU/ml), while C-peptide was within the reference range (681 pmol/L) and proinsulin was below normal (2.7 mmol/L) as well as beta-hydroxybutyrate (27.8 mmol/L). Anti-insulin antibody titer (IgG) was significantly elevated (>100.0 IU/mL).

Other laboratory investigation results (Table 2), including those of full blood count, liver function test, renal function test, and thyroid profile, were normal. Adrenocorticotropic hormone (ACTH) and nonstimulated morning cortisol levels were normal. ACTH stimulation test was not done as features of hyperinsulinism were evident on laboratory results during the hypoglycaemic event. Total testosterone and luteinising hormone (LH) levels, tested one week before admission by the out-patient clinic, were below the reference range (4.2 nmol/L [reference range 6.1–27.1 nmol/L] and 0.7 IU/L [reference range 1.24–8.68 IU/L], respectively). This could be explained by his previous use of recreational anabolic steroids. The prolactin level was only mildly elevated (317 mIU/L [reference range 57–281 mIU/L]), and follicular stimulating hormone (FSH) level was within reference range (1.6 IU/L [reference range 1.27–19.26 IU/L]). Magnetic resonance imaging (MRI) of the abdomen, including the pancreas, and the *sella turcica*, showed no abnormalities.

Given the high levels of anti-insulin antibodies, hyperinsulinaemia, late postprandial hypoglycaemia, and clinical presentation in coincidence with intake of anabolic steroids and other products like thiol and amino acids, and with exclusion of other differential diagnoses of hypoglycaemia, anti-insulin antibodies-induced hypoglycaemia syndrome, also called Insulin autoimmune syndrome (IAS), was the most likely explanation of patient's condition and continued hypoglycaemia.

## 3. Treatment

The patient was started on a high dose of glucocorticoid therapy with prednisolone 1 mg/kg/day while in the medical ward. The patient then travelled abroad less than a month later for further medical work-up and care in the United States (US) before his planned follow up visit in the endocrine clinic. A similar diagnosis of IAS was made based on the same work-up done at our hospital while no further hypoglycaemic events occurred, and the patient was kept on prednisolone (1 mg/kg/day) and was asked to continue the therapy for several weeks with tapering dose until discontinuing it.

## 4. Outcome and Follow Up

**Figure 1:**
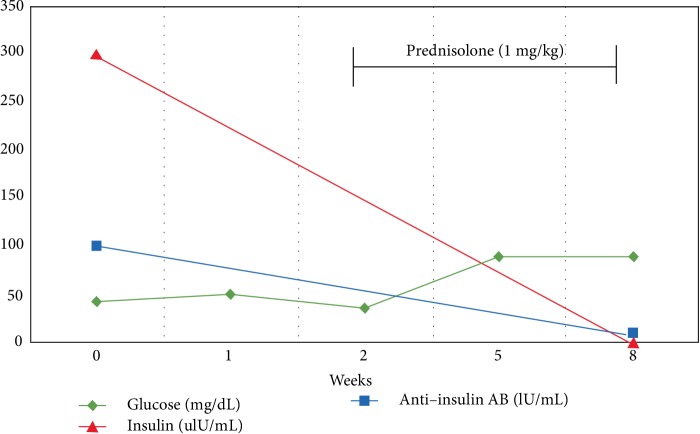
Resolution of hypoglycaemia and anti-insulin antibodies after starting prednisolone in our patient.

The patient returned to Kuwait after the two weeks visit to the US and then attended the endocrine clinic after 5 weeks of therapy with prednisolone. The patient never had any further hypoglycaemic attack during the course of therapy and was asked to start tapering down the dose at that point. After discontinuing the glucocorticoid therapy, the patient never developed any hypoglycaemia symptoms indicating remission of the condition. His last anti-insulin antibody titer test was done in July 2014 with negative results (Figure 1).

## 5. Discussion

IAS, or Hirata disease, is a rare cause of endogenous hyperinsulinaemic hypoglycaemia, characterized by autoantibodies to endogenous insulin in individuals without previous exposure to exogenous insulin [1]. The condition is known as Hirata's disease (HD), after Yukimasa Hirata, the author who first described the syndrome in 1970 [2]. It is now recognized as the third most common cause of hypoglycaemia in Asian patients [3] following insulinoma and nonpancreatic neoplasia as the first and second most common aetiologies, respectively [3]. The syndrome affects both sexes equally [4] and is more frequent in patients over 40 years of age. Interestingly, our patient was much younger, and this condition is rarely reported among children or at a young age.

The mechanism of hypoglycaemia in IAS is unknown but the most widely accepted hypothesis is a mismatch between blood glucose and free insulin concentration, secondary to the binding and release of secreted insulin by autoantibodies [5]. Usually, after a meal, glucose concentration rises which stimulates insulin secretion. In this disorder, autoantibodies bind to insulin molecules and inhibit their effect, leading to hyperglycaemia [4, 5]. As glucose concentration eventually falls, insulin secretion also subsides, and the total insulin level decreases. At this time, insulin molecules spontaneously dissociate from the autoantibodies giving rise to a high free insulin level inappropriate for the glucose concentration, thus provoking hypoglycaemia [5]. Due to this insulin-glucose mismatch, on several occasions insulinoma is suspected instead of IAS [6]. When insulin autoantibodies bind to insulin, the half-life increases from 5 minutes to hours [7], while the half-life of the C-peptide usually remains shorter and unaffected (30−35 minutes) giving rise to an elevated insulin level while having a nonelevated C-peptide and proinsulin and changing the insulin to c-peptide ratio to >1. It is also possible that the insulin autoantibody is capable of binding to endogenous proinsulin and C-peptide as well, delaying their half-lives, and possibly keeping the insulin to C-peptide ratio in the normal range of <1 [8, 9]. In our patient, the proinsulin was below reference range indicating an inhibitory effect on the proinsulin synthesis due to the hyperinsulinaemic period or a possible nonextended half-life of proinsulin which helped to clear it before the onset of hyperinsulinaemia. Furthermore, the C-peptide level was within the reference range, indicating a similar nonextended half-life with possible insulin to C-peptide ratio >1, but the insulin level could not be measured beyond 301 uIU/mL.

Also, high serum insulin levels can be accompanied by low proinsulin and C-peptide levels, misleading the diagnosis as injectable insulin administration [3]. Our patient denied using exogenous insulin while proinsulin was below the reference range and the C-peptide level was normal.

The anti-insulin antibody could not be detected beyond 100 IU/mL. In case of recurrence, further detailed evaluation can be conducted by sending the antibody for laboratory testing abroad.

IAS has an association with other autoimmune diseases such as Graves' disease, rheumatoid arthritis, and HLA DR4, which has been demonstrated in 96% of Japanese patients with IAS [1]. The most common drug associated with IAS is methimazole, while the use of other drugs such as carbimazole, glutathione, tiopronin, tolbutamide, gold thioglucose, interferon-*α*, captopril, diltiazem, hydralazine, procainamide, isoniazid, D-penicillamine, imipenem, and penicillin has been associated with this condition [4, 5, 10]. A few case reports of IAS associated with the use of alpha lipoic acid (ALA) were also reported [10, 11]. Also, insulin autoantibodies may be triggered by exposure to viruses like mumps, rubella, Coxsackie B influenza, hepatitis C, chickenpox, and measles by acting as super-antigens or they may manifest spontaneously [3].

Our patient had a history of anabolic steroids, growth hormone, and oral amino acids use which may have contributed to his condition. Further information on the supplements used by the patient was not known; such information would have helped us in evaluating this case since some health supplements have been reported to be triggers for IAS. Furthermore, our patient did not have any previous exposure to exogenous insulin. To our knowledge, this is the first documented case of IAS in a young healthy male without any associated condition or autoimmune disease in the Arabian Gulf region.

Although a spontaneous improvement of the symptoms was reported in the literature [4], most patients were treated differently by various investigators [4]. Management of IAS as reported by different case reports included using different immunosuppressive regimens, such as prednisolone [12], hydrocortisone [13], cyclophosphamide [14], azathioprine [15], rituximab [16], mycophenolate mofetil [17], and plasmapheresis [18, 19]. Our patient had a complete recovery after 8-weeks course of prednisolone, although many case reports documented a recovery period after 12-weeks of prednisolone [20, 21].

IAS could be a misleading condition and careful diagnosis is needed to rule out other causes of hyperinsulinaemic hypoglycaemia, especially in patients with previous history of bariatric surgery or signs of insulinoma. The patient history of supplements intake and use of recreational hormones is important in the evaluation of IAS as precipitating factors with the possibility of additional unknown triggers.

## Figures and Tables

**Table 1:** Results of 72-hour fasting test.

Symptoms of hypoglycaemia	Yes
Glucose (reference range 3.9–6.1 mmol/L)	2.4 mmol/L
Insulin (reference range 1.9–23 uIU/ml)	>301 uIU/mL
C-peptide (reference range 33–1458 pmol/L)	681 pmol/L
Proinsulin (reference range <11.0 pmol/L)	2.7 pmol/L
Beta-hydroxybutyrate (reference range 30–300 mmol/L)	27.8 mmol/L
Circulating oral hypoglycaemic agents	Not available
Antibodies to insulin IgG (reference range 0–10 IU/ml) by enzyme-linked immunosorbent assay (ELISA)	>100.0 IU/mL

**Table 2:** Other laboratory investigations.

Test	Result	Reference range
Haemoglobin A1c	5.1%	Nondiabetic (<5.7%)
Anti-GAD Ab	Negative	0–10 IU/mL
Anti-IA2 Ab	Negative	0–10 IU/mL
Anti-islet cells Ab	Negative	0–10 IU/mL
Insulin-like growth factor 1 (IGF-1)	33	29–85 nmol/L (adults 20–30 years)
Cortisol morning	467	185–624 nmol/L
Cortisol evening	255	150–276 nmol/L
Adrenocorticotropic hormone (ACTH)	42.3	10–46 pg/mL
LH	0.7	1.24–8.68 IU/L
FSH	1.6	1.27–19.26 IU/L
Prolactin	317	57–281 mIU/L
Total testosterone	4.2	6.1–27.1 nmol/L
